# Efficacy and safety profile of linezolid in the treatment of multidrug-resistant (MDR) and extensively drug-resistant (XDR) tuberculosis: a systematic review and meta-analysis

**DOI:** 10.1186/s12941-016-0156-y

**Published:** 2016-06-22

**Authors:** Akosua Adom Agyeman, Richard Ofori-Asenso

**Affiliations:** Research Unit, Health Policy Consult, Weija, P. O. Box WJ 537, Accra, Ghana

**Keywords:** Linezolid, Tuberculosis, Multi-drug resistance, Extensively drug resistant, Meta-analysis, Drug therapy, Infectious diseases

## Abstract

**Background:**

Treatment options for drug-resistant tuberculosis are still limited. Linezolid has been recommended for treatment of patients with multidrug-resistant (MDR) or extensively-drug-resistant (XDR) tuberculosis, although uncertainties remain regarding its safety and tolerability in these circumstances.

**Objective:**

To systematically evaluate the existing evidence regarding the efficacy and tolerability of linezolid in the treatment of MDR or XDR tuberculosis.

**Methods:**

We conducted a systematic review and meta-analysis in accordance with the PRISMA guidelines. Searches were conducted in PubMed, Web of Science and EMBASE followed by direct search of abstracts in the International Journal of Tuberculosis and Lung Disease to retrieve primary studies published between January 2000 and January 2016 assessing linezolid efficacy and safety in the treatment of drug-resistant TB. We evaluated the occurrence of outcomes including culture conversion, treatment success and incidence of adverse events such as myelosuppression and neuropathy.

**Results:**

Twenty-three (23) studies conducted in fourteen (14) countries and involving 507 patients were retrieved. Only 1 randomized controlled trial was identified and none of the identified studies involved participants from Africa. The pooled proportion for treatment success was 77.36 % (95 % CI = 71.38–82.83 %, I^2^ = 37.6 %) with culture conversion rate determined as 88.45 % (95 % CI = 83.82–92.38 %, I^2^ = 45.4 %). There was no strong evidence for both culture conversion (p = 0.0948) and treatment success (p = 0.0695) between linezolid daily doses ≤ 600 and > 600 mg. Only myelosuppression showed a strong statistical significance (p < 0.0001) between dose comparisons. The incidence of neuropathy and other adverse events leading to permanent discontinuation of linezolid also showed no significance upon dose comparisons (p = 0.3213, p = 0.9050 respectively).

**Conclusion:**

Available evidence presents Linezolid as a viable option in the treatment of MDR/XDR TB although patients ought to be monitored closely for the incidence of major adverse events such as myelosuppression and neuropathy. Additionally, highly powered randomized controlled trials including participants from endemic regions are urgently needed to better inform the magnitude and significance of Linezolid treatment effect in MDR and XDR TB patients.

**Electronic supplementary material:**

The online version of this article (doi:10.1186/s12941-016-0156-y) contains supplementary material, which is available to authorized users.

## Background

Tuberculosis (TB) is a significant contributor to global morbidity and mortality. About one in three persons representing almost 3 billion individuals worldwide are known to be infected with *Mycobacterium tuberculosis* of which at least 5 % are likely to develop active TB disease during their lifetime [1, 2]. In 2014, more than 9 million new cases of TB were recorded resulting in over 1.5 million deaths [2]. Nearly one in three deaths in HIV-positive individuals are attributable to TB [3]. Disproportionate number of global TB cases are known to occur in areas such as Sub-Saharan Africa and South East Asia [2]. The economic impact of TB is deemed to be enormous as more than 90 % of TB-related deaths occur among adults in the most productive years [4].

Over the last few years, significant progress has been made towards controlling TB and reducing the global burden of the disease. TB incidence has declined in all parts of the world by at least 1.5 % annually since 2000 and is now almost 18 % lower than the rate in 2000 [1, 5]. Additionally, TB mortality has decreased by almost 50 % since 1990, with nearly all of that improvement happening in the era of the millennium development goals (MDGs) [5]. In the context of these TB control successes, it is estimated that over 40 million lives were saved in the period 2000–2014 [1].

However, in spite of the positive developments, the increasing emergence of multidrug-resistant (MDR) and extensively drug-resistant (XDR) TB across the globe has the potential to derail the fight against TB and possibly revert the progress made regarding TB care and control. MDR-TB has been used to represent all forms TB disease in which the causative bacteria is resistant to at least isoniazid and rifampicin, whereas XDR-TB denotes forms of TB in which the bacteria is resistant to rifampicin and isoniazid plus any fluoroquinolone, and at least one of the second line injectable TB drugs (i.e., amikacin, kanamycin, or capreomycin) [6]. By the end of 2013, over 90 countries had documented at least a case of XDR-TB [7]. Almost 5 % of all global TB cases are now estimated to be MDR-TB including over 3 % of newly diagnosed TB cases, and as much as 20 % in previously treated patients [8, 9]. In 2014 alone, more than 400,000 cases of MDR-TB were reported with nearly 10 % of this being XDR-TB [8].

The cost implication of MDR/XDR TB is enormous and one that could impose significant strain on any healthcare system. Diel et al., for instance, estimated the total cost per MDR-TB and XDR-TB case in Germany to be €82,150 and €108,733, respectively [10]. Within the period 2011–2015, as much as USD 1.7 billion was required across the world in tackling MDR-TB [11].

Treatment outcomes for MDR/XDR-TB remain poor even in advanced health systems. In 2007, the World Health Organization (WHO) reported that just around one-third of the over 7000 MDR-TB patients from 13 countries were successfully treated [12]. On the other hand, for nearly four decades no new anti -tubercular drug was registered until the recent introduction of Delamanid and Bedaquiline [13, 14]. Even these new drugs are unable to resolve all the challenges regarding therapy for MDR/XDR-TB [14]. In view of this, diverse treatment approaches have continually been explored including the use of therapies containing linezolid, higher doses of isoniazid and sometimes fluoroquinolones [15].

Linezolid an Oxazolidinone and a relatively newer class of antibiotic has demonstrated potency against drug-resistant *M. tuberculosis* in a number of in vitro studies [16–18]. Since 2006, the WHO has recommended the use of linezolid in the treatment of MDR/XDR-TB with the drug now being included in many TB programmes across the world [19, 20]. Aside its high cost which remains a major barrier to access, there are uncertainties regarding the most effective dose of linezolid with < 600 or ≥ 600 mg daily doses being documented in separate reports [19, 21]. Additionally, serious adverse effects such as neuropathies and hematological adverse reactions have been reported raising huge concerns about the safety of the drug in the treatment of XDR and MDR-TB which usually demands extensive treatment periods [19, 21].

Some reviews have previously sought to assess the efficacy and tolerability/safety of linezolid in the treatment of MDR/XDR-TB [19–23]. The latest of these reviews conducted by Zhang et al. [23], includes primary studies published no later than May 2014. Considering that scientific evidence changes rapidly and according to Whitlock et al. [24], reviews are deemed to be out of date often after few years, there is the need for continuous evaluation of evidence to incorporate new information as they become available. In view of this, we conducted a systematic review and meta-analysis to summarize the existing evidence to date of the safety and efficacy of linezolid in the treatment of DR-TB as an update to previously conducted reviews.

## Methods

This systematic review was conducted in accordance with the PRISMA (preferred reporting items for systematic reviews and meta-analyses) guidelines [25].

### Search strategy and study selection

We performed searches in PubMed, Web of Science and EMBASE for relevant studies published between January 2000 and January 2016. In addition, we searched the International Journal of Tuberculosis and Lung Disease for original studies published on the subject within the above period. A combination of key words and their synonyms used in all searches were ‘multidrug resistant tuberculosis’, ‘extensively drug resistant tuberculosis’, ‘linezolid’, ‘zyvox’, ‘efficacy’ and ‘toxicity’. We included ‘zyvox’ as a keyword as it is the most commonly marketed brand name for linezolid [26]. Search results were limited to human population and English language. References of selected studies and previously published reviews were also screened to identify additional publications. We included only published primary studies involving adult populations of ≥5 patients with sputum culture confirmed (pulmonary or extra pulmonary TB) and available report on efficacy and tolerability (safety). In vitro studies and review articles were excluded as well as case reports with sample size less than 5 patients. Exclusion of  studies with relatively small sample size was intended to minimize selection and reporting bias [27].

### Study quality assessment

We employed the McMaster critical review for quantitative studies to critically appraise all studies [28, 29]. Further methodological quality assessments of studies were conducted based on the following criteria: linezolid dose stated, DST guided treatment regimen, hospitalization at initiation of linezolid treatment, IRB approval obtained, patients monitored by DOT and outcomes report similar to WHO definitions.

### Data extraction

A data extraction form was developed and used to guide the extraction of data from the included studies. First author name, publication year, duration of study, type of study design, control group present, country of study and number of HIV co-infected patients were extracted for epidemiological characteristics. With regards to efficacy and tolerability, data extracted included total number of MDR/XDR TB exposed to linezolid, treatment regimen employed and linezolid dose. Outcome measures on efficacy and tolerability were based on WHO definitions [30]. For efficacy, data were extracted for patients who achieved sputum culture conversion to negative as well as those who were cured. Patients who defaulted, achieved treatment failure, relapsed or died were regarded as unfavorable outcomes. For tolerability, data were extracted for neuropathy, myelosuppression, both temporary and permanent discontinuation of linezolid due to adverse effects and other reported adverse effects associated with linezolid [31]. A summary of the data for outcomes evaluation has been provided as a supplementary material (Additional file 1). All data were extracted by AA and verified by RO. Where there were disagreements, these were resolved by consensus-based discussions.

### Statistical analysis

The meta-analysis proportions were conducted using StatsDirect statistical software (Version 3.0.0, StatsDirect Ltd, Cheshire UK) [32]. Individual study proportions were assessed at 95 % confidence interval (CI) as well as the pooled effect. Between-study heterogeneity was assessed by the Quoran (Q) statistic test and the *I*^2^ statistic, which represents the percentage of total variation across studies, attributable to heterogeneity rather than to chance [33]. As we anticipated variations among studies for multiple reasons including study conduct methods, the random effect model (DerSimonian-Laird) was adopted over fixed effect model in the summary of pooled analysis [33]. Publication bias was evaluated by direct observation of  funnel plots and the Egger and Begg’s tests were applied to measure any asymmetry [34]. For all computations statistical significance was set at p < 0.05.

## Ethical approval

Ethical approval was not sought for this study as all information used were derived from already published studies available in the public domain.

## Results

### Studies identification and retrieval

**Fig. 1:**
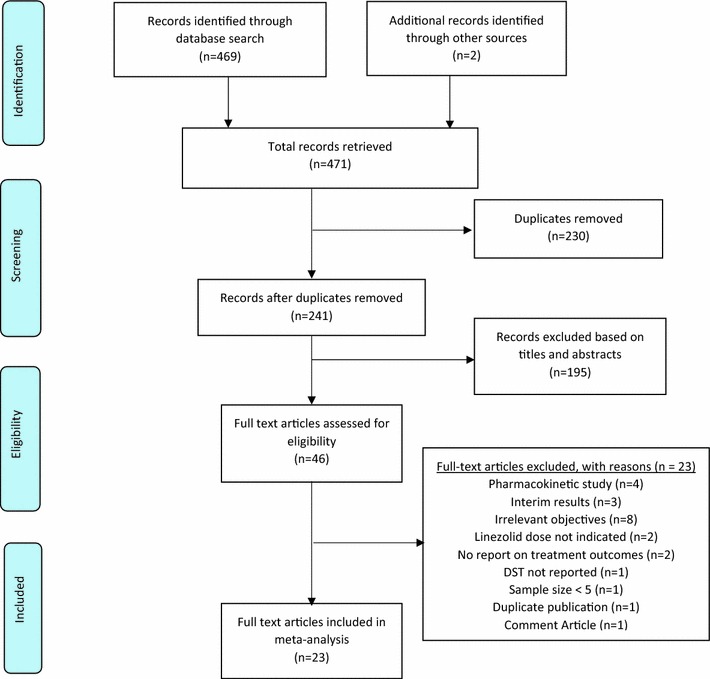
A schematic flow diagram of studies’ search and retrieval process

A total of 469 records were retrieved from database search in addition to two records identified through International Journal of Tuberculosis and Lung Disease. Upon removing duplicates and screening by titles and abstracts, 46 articles were found relevant for full-text analysis and reference list screening. Subsequent to this, 23 articles were excluded with reasons (Fig. 1) and 23 studies were identified as eligible for inclusion in the meta-analysis [35–57]. The 23 studies were conducted in 14 countries across the globe. Per regional distribution, more than half (57 %, n = 13), were conducted in Asia. The rest of the studies were conducted in North America (n = 4), South America (n = 1) and Europe (n = 5). None of the selected studies was conducted in Africa. About 57 % (n = 13) of studies were conducted in the last 5 years (2011–2016). Most of the studies were case series (n = 20, 87 %). Only one randomized controlled trial was identified [51]. The two remaining studies consisted of one non-randomized Phase 1 clinical trial [36] and the other a Phase 2a clinical trial [42]. A total of 507 patients received linezolid as part of their treatment regimen and 353 patients were evaluated for definite outcomes (cured, treatment completed, died, failure). About 57 % of patients enrolled tested positive for XDR TB and 3 % had documented HIV positive status (Table 1). Thus the population involved was predominantly HIV negative. In most of the studies, linezolid was included in the treatment regimen based on DST following treatment failure to previous treatment regimen. Linezolid was generally administered at a daily minimum dose of 300 mg to a maximum dose of 1200 mg. The duration of treatment ranged from 1 to 36 months. The quality assessment across studies was generally satisfactory. All studies indicated linezolid dose and treatment regimen was individualized based on DST results (Table 2). However, 19 out of 23 studies reported Institutional Review Board (IRB) approval prior to study initiation. The remaining four studies did not report on IRB approval [38, 43, 45, 58]. Hospitalization prior to linezolid treatment was poorly reported; no reporting was done by 15 studies. Nonetheless, 6 studies reported patient hospitalization prior to linezolid treatment and only 2 studies indicated no patient hospitalization. Also, DOT was not reported in 12 out of 13 studies. With respect to treatment success, sixteen (16) studies were similar to WHO definitions whiles four (4) studies did not conform to WHO standards and three (3) studies did not report on their reference guideline.

**Table 1:** Description of the characteristics of included studies

Study No.	Reference	Year of publication	Country of study	Study design	Control group	Number exposed to Linezolid	Study duration	Number of XDR-TB	LZD dosage	Duration of LZD treatment	Type of anti-TB regimen	HIV infection status
1.	Abbate et al.[35]	2012	Argentina	Retrospective study	No	17	2002–2008	17	600 mg bd	≥12 months after cc	Individualized	All HIV negative
2.	Anger et al. [36]	2010	USA	Retrospective case series	No	16	2000–2006	10	600 mg bd 400 mg bd or 600 mg daily	Mean = 15 Median = 16 Range = 1–29 months	Individualized	3 HIV positive
3.	Condos et al.[37]	2008	USA	Prospective phase 1 clinical trial	No	6^a^	2000–2007	6^a^	600 mg bd 600 mg daily	Range = 9–26 months	Individualized	All HIV negative
4.	De Lorenzo et al. [38]	2012	Italy	Retrospective study	No	12	2009–2010	4	600 mg bd (10 patients) 600 mg daily (1 patient) 450 mg bd (1 patient)	Range = 37–100 days Median = 63.5 days	Individualized	2 HIV negative
5.	Fortún et al. [39]	2005	Spain	Retrospective case series	No	3^b^	1994–2004	0	600 mg bd	Range = 4–24 months Mean = 12 Median = 12	Individualized	1 HIV positive
6.	Koh et al. [40]	2009	South Korea	Retrospective case series	No	24	2007–2008	1	300 mg daily	Median = 12 months	Individualised	All HIV Negative
7.	Koh et al. [41]	2012	South Korea	Retrospective case series	No	51	2007–2009	26	300 mg daily	Median = 413 days IQR = 237–622 days	Individualized	All HIV negative
8.	Lee et al. [42]	2012	South Korea	Phase 2a randomized two-group study	No	38	2008–2011	41	600 mg daily	22 months	Individualized	All HIV negative
9.	Liu et al. [43]	2015	China	Retrospective case series	No	16	2011–2013	16	600 mg daily	Range = 3–21 months Mean = 9.53 months	Individualized	All HIV negative
10.	Migliori et al. [44]	2009	Belarus, Germany, Italy, Switzerland	Retrospective non-randomized unblinded observational study	Yes	85	2001–2007	12	600 mg daily 600 mg bd	Mean = 7 months Median = 3 months	Individualized	3 HIV positive
11.	Nam et al. [45]	2009	South Korea	Retrospective case series	No	11	NR	4	600 mg daily 300 mg bd	Range 3–24 months Mean = 7 months Median 5 months	Individualized	All HIV negative
12.	Park et al. [46]	2006	South Korea	Prospective non-randomized case series	No	8	2003–2006	5	600 daily 600 bd	Range = 3–18 months Median = 9 months Mean = 11 months	Individualized	All HIV negative
13.	Roongruangpitayakul et al. [47]	2013	Thailand	Retrospective case series	No	24	2009–2012	7	600 mg daily 300 mg daily	Range = 11.0–21.5 months Mean = 18.7 months	Individualized	All HIV negative
14.	Schecter et al. [48]	2010	USA	Retrospective case series	No	30	2003–2007	3	600 mg daily	Range = 1–36 months Median = 22 months Mean = 19 months	Individualized	17 HIV negative
15.	Singla et al. [49]	2012	India	Prospective case series	No	29	2006–2011	16	600 bd 600 daily	Median = 30 days	Individualized	All HIV negative
16.	Tang et al. [50]	2011	China	Case series	No	14	2009–2010	14	600 mg bd 600 mg daily	Range= 2–11 months Mean: 6.5 months	Individualised	All HIV negative
17.	Tang et al. [51]	2015	China	Prospective multicenter randomized controlled study	Yes	33	2009–2011	65	1200 mg daily 300–600 mg daily	Range = 2–24 months	Individualized	All HIV negative
18.	Tse-Chang et al. [52]	2013	Canada	Retrospective case study	No	13	2000–2011	NR	600 mg daily	Mean = 8.3 months Range = 1.4–22 months	Individualized	1 HIV positive
19.	Udwadia et al. [53]	2010	India	Prospective non-randomized case series	No	18	2000–2007	7	600 mg daily	Mean = 21 months	Individualized	NR
20.	Villar et al. [54]	2011	Portugal	Prospective case series	No	16	2004–2009	12	600 daily 1200 mg daily	Median = 375 days	Individualized	6 HIV positive
21.	Von der Lippe et al. [55]	2006	Norway	Retrospective case series	No	10	1998–2002	0	600 mg bd	Range = 2–10 months Median = 4.25 months	Individualized	1HIV positive
22.	Xu et al. [56]	2012	China	Retrospective case series	No	18	2007–2010	15	600 mg bd 900 mg daily	Range =1.5–10 months Median = 6 months	Individualized	All HIV negative
23.	Zhang et al. [57]	2014	China	Retrospective study	Yes	15	2012–2013	43	600 mg daily	Range = 1–5 months	Individualized	All HIV negative

^a^ One paediatric case excluded

^b^ Excluded two patients with *M. bovis* infection

**Table 2:** Summary of the methodological quality assessment of included studies

Study No.	References	IRB approval	LZD dose indicated	Individualised treatment based on DST	Hospital admission prior to LZD treatment	DOT during treatment	Treatment success definition similar to WHO
1.	Abbate et al. [35]	Yes	Yes	Yes	NR	NR	Yes
2.	Anger et al. [36]	Yes	Yes	Yes	NR	Yes	Yes
3.	Condos et al. [37]	Yes	Yes	Yes	NR	NR	Yes
4.	De Lorenzo et al. [38]	NR	Yes	Yes	NR	NR	Yes
5.	Fortún et al. [39]	Yes	Yes	Yes	NR	Yes	Yes
6.	Koh et al. [40]	Yes	Yes	Yes	NR	Yes	Yes
7.	Koh et al. [41]	Yes	Yes	Yes	NR	NR	Yes
8.	Lee et al. [42]	Yes	Yes	Yes	Yes	Yes	No
9.	Liu et al. [43]	NR	Yes	Yes	Yes	NR	NR
10.	Migliori et al. [44]	Yes	Yes	Yes	NR	Yes	Yes
11.	Nam et al. [45]	NR	Yes	Yes	NR	NR	No
12.	Park et al. [46]	Yes	Yes	Yes	NR	NR	Yes
13.	Roongruangpitayakul et al. [47]	Yes	Yes	Yes	No	Yes	Yes
14.	Schecter et al. [48]	Yes	Yes	Yes	NR	Yes	Yes
15.	Singla et al. [49]	Yes	Yes	Yes	Yes	Yes	No
16.	Tang et al. [50]	Yes	Yes	Yes	NR	NR	NR
17.	Tang et al. [51]	Yes	Yes	Yes	NR	Yes	Yes
18.	Tse-Chang et al. [52]	NR	Yes	Yes	NR	NR	Yes
19.	Udwadia et al. [53]	Yes	Yes	Yes	No	NR	NR
20.	Villar et al. [54]	Yes	Yes	Yes	NR	NR	Yes
21.	Von der Lippe et al. [55]	Yes	Yes	Yes	Yes	Yes	No
22.	Xu et al. [56]	Yes	Yes	Yes	Yes	Yes	Yes
23.	Zhang et al. [57]	Yes	Yes	Yes	Yes	NR	Yes

### Efficacy

**Fig. 2:**
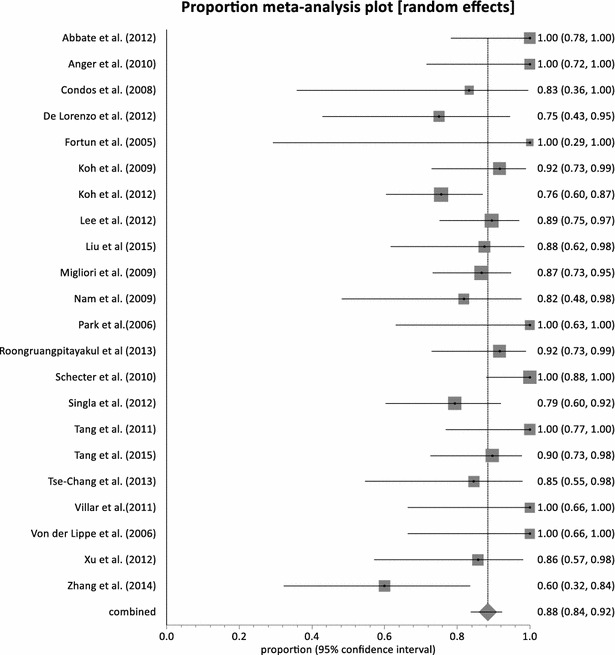
Forest plot of culture conversion, individual and pooled (random effects)

**Fig. 3:**
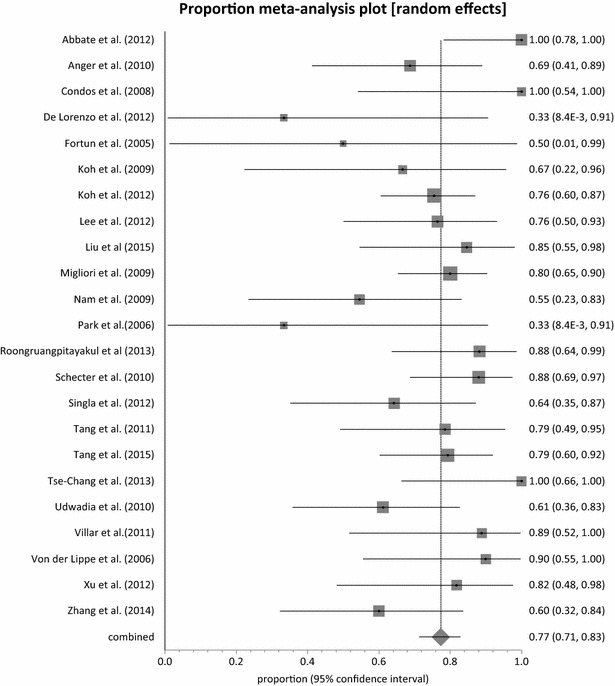
Forest plot of treatment success, individual and pooled (random effects)

With the exception of Udwadia et al. [53], all the studies reported on sputum culture conversion with a pooled proportion of 88.45 % (95 % CI = 83.82–92.38 %, p = 0.0112) (Fig. 2) and moderate heterogeneity across studies (I^2^ = 45.4 %; 95 % CI = 0–65.9 %). Eight studies [35, 36, 39, 46, 48, 50, 54, 55], achieved 100 % sputum culture conversion with a total number of 98 out of 507 patients exposed to linezolid. Among these eight studies, three studies [35, 39, 55] administered linezolid at a dose of 600 mg twice daily with only one study administering at a dose of 600 mg daily. The remaining four studies had mixed dosing regimen in the same cohort of patients. A total of 274 patients achieved treatment success across the 23 studies with a combined proportion of 77.36 % (95 % CI = 71.38–82.83 %, p = 0.0365) (Fig. 3) and a low homogeneity test result of 37.6 % (95 % CI = 0–61.3 %). Only two studies [38, 46] had less than 50 % treatment success with linezolid dose regimen between 600 and 1200 mg daily. Three studies [35, 37, 52] reported 100 % treatment success (95 % CI = 78.20–100, 54.07–100 and 66.37–100 %, respectively).

### Safety and tolerability

**Fig. 4:**
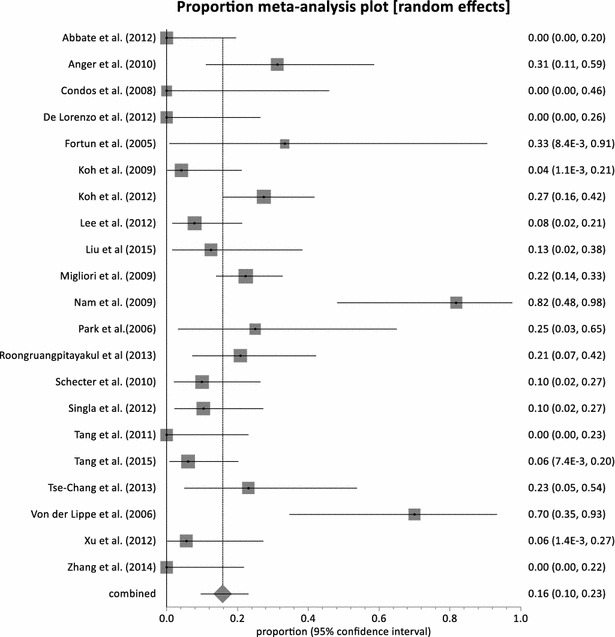
Forest plot of discontinuation due to linezolid adverse effects, individual and pooled (random effects)

**Fig. 5:**
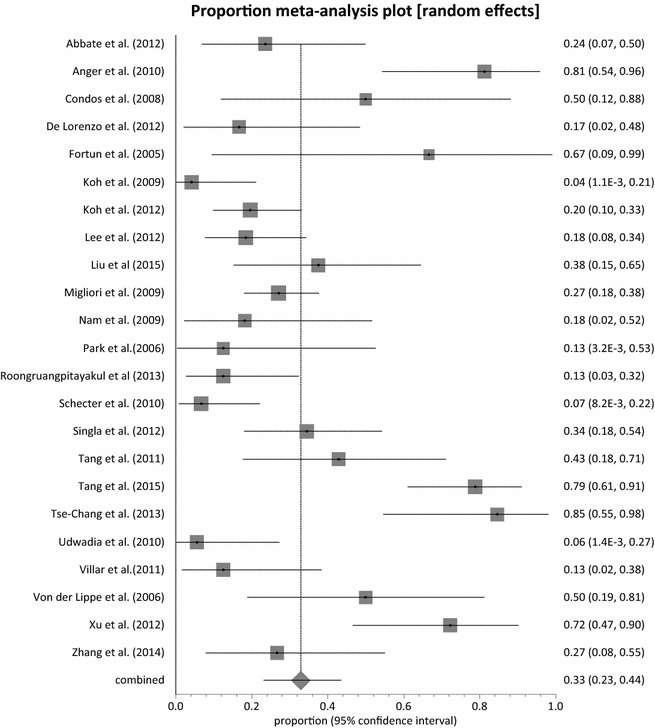
Forest plot of reported myelosuppression, individual and pooled (random effects)

**Fig. 6:**
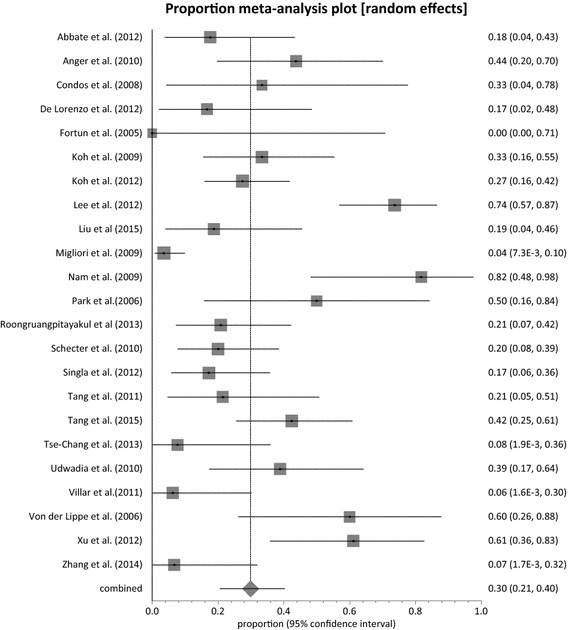
Forest plot of reported neuropathy, individual and pooled (random effects)

**Fig. 7:**
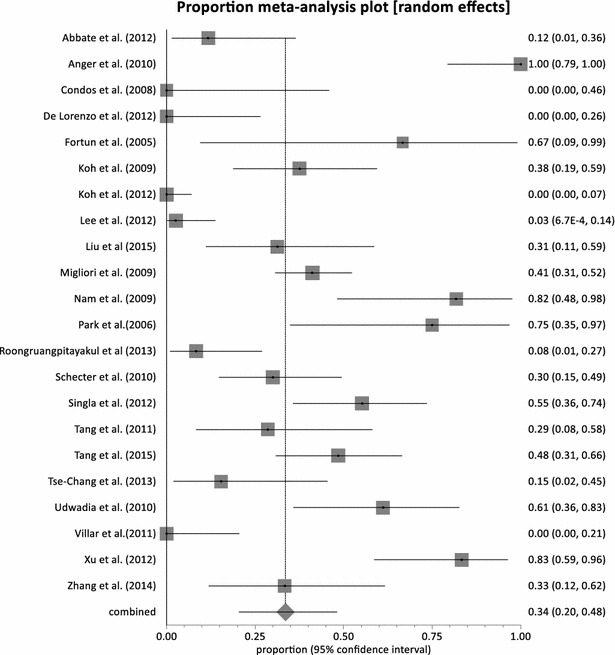
Forest plot of reported other adverse events, individual and pooled (random effects)

Adverse events related to Linezolid was observes in all the studies. Major adverse events leading to permanent discontinuation of linezolid was observed in 21 studies with pooled proportion of 15.81 % (95 % CI = 9.68–23.11 %, p < 0.0001) (Fig. 4). Heterogeneity was observed to be very high at 74 % (95 % CI = 58.0–82.0 %). Two studies did not report whether any permanent discontinuation due to linezolid toxicity had happen or not [53, 54]. On the other hand, in five studies there was no occurrence of permanent discontinuation of linezolid due to adverse events in patients [35, 37, 38, 50, 57]. All 23 studies reported myelosuppression in the form of anemia or neutropenia. The pooled proportion of myelosuppression was observed at 32.93 % (95 % CI = 23.13–43.54 %, p < 0.0001) (Fig. 5) and high heterogeneity of 83 %. The Canadian cohort [52] recorded the highest incidence of myelosuppression of 85 % (11 out 13 patients) at a linezolid dose of 600 mg daily. Koh et al. [40] recorded the least occurrence of myelosuppression; 4 % (1 out of 24 patients) with daily 300 mg dose of linezolid. Neuropathy was also recorded in all but one studies with a combined proportion of 29.92 % (95 % CI = 20.53–40.25 %, p < 0.0001) (Fig. 6). None of the patients enrolled in Fortun et al. experienced neuropathy and Linezolid was given at a dose of 600 mg BD [39]. The highest proportion of neuropathy was observed in Nam et al. [45] with a proportion of 81.82 % (95 % CI = 48.22–97.72 %) where linezolid was administered at a maximum dose of 600 mg daily. With the exception of Von der Lippe et al. [55], adverse events other than myelosuppression and neuropathy were reported in the remaining 22 studies. Nausea and vomiting were the most frequently reported. Others included hyperpigmentation of the oral cavity [51] and transient visual impairment [47]. The pooled proportion of reported adverse events other than myelosuppression and neuropathy was 33.60 % (95 % CI = 20.41–48.23 %, p < 0.0001) (Fig. 7).

### Outcomes comparison between daily doses ≤ 600 and > 600 mg

Patients receiving linezolid at a dose ≤ 600 mg had lower proportions (85.58 %) of culture conversion compared to those receiving linezolid at doses > 600 mg (95.12 %). There was no strong evidence for both culture conversion (p = 0.0948) and treatment success (p = 0.0695) between linezolid doses ≤ 600 and > 600 mg (Table 3). Nonetheless, higher proportion of patients achieved treatment success in higher doses of linezolid (89.47 %) compared to administering lower doses of linezolid (76.14 %). Linezolid doses > 600 mg observed higher incidence of myelosuppression (50 %) compared with doses ≤ 600 mg (19.58 %). Only myelosuppression showed a strong statistical significance (p < 0.0001) between dose comparisons. On the contrary, the incidence of neuropathy and adverse events leading to permanent discontinuation of linezolid showed no significance upon dose comparisons (p = 0.3213, p = 0.9050 respectively).

**Table 3:** Comparison of treatment outcomes of MDR/XDR-TB cases according to daily administered linezolid dose

Outcome	≤600 mg linezolid n (%)	>600 mg linezolid n (%)	Difference (%) (95 % CI)	p value
Culture conversion	184/215 (85.58)	39/41 (95.12)	9.54 (−2.29–16.32 %)	p = 0.0948
Treatment success	134/176 (76.14)	34/38 (89.47)	13.34 (−13.22–23.12 %)	p = 0.0695
Myelosuppression	47/240 (19.58)	24/48 (50.00)	30.42 (15.77–44.94 %)	p < 0.0001
Neuropathy	82/240 (34.17)	20/48 (41.67)	7.5 % (−6.84–22.79 %)	p = 0.3213
Linezolid discontinuation	40/222 (18.02)	9/48 (18.75)	0.73 % (−9.66–14.72 %)	p = 0.9050

### Publication bias

**Fig. 8:**
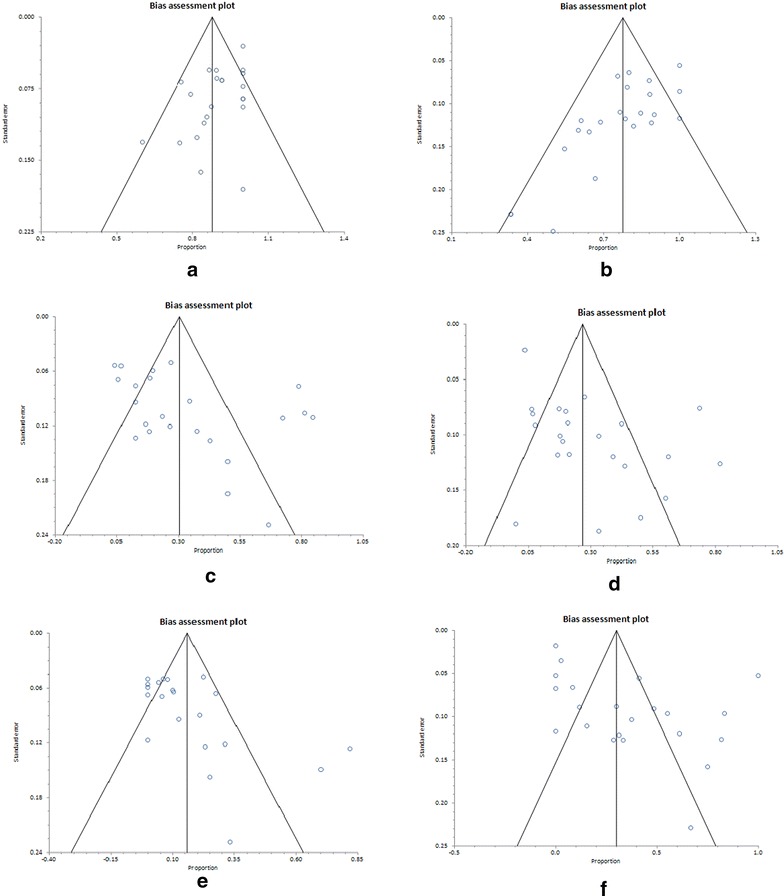
Bias assessment (funnel) plots of publication bias for the outcomes evaluated (**a** culture conversion; **b** treatment success; **c** myelosuppression; **d** neuropathy; **e** discontinuation due to linezolid adverse effects; **f** presence of any other adverse events)

Begg’s and Egger’s regression tests were performed to assess publication bias. The shapes of the funnel plots do not show obvious evidence of asymmetry (Fig. 8). However, the *p* value of Egger’s test confirmed the existence of publication bias for all the outcomes evaluated [(A) Culture conversion, p = 0.0144; (B) treatment success, p = 0.0006; (C) myelosuppression, p = 0.0295; (D) neuropathy p = 0.0014; (E) discontinuation due to linezolid adverse effects, p = 0.01 and (F) presence of any other adverse events, p = 0.0067].

## Discussion

This systematic review and meta-analysis included a larger number of case reports and observational studies than reported in previous reviews which suggests that linezolid is increasingly being used off-label in the management of drug resistant TB. In our systematic review, only one randomized controlled trial (with ‘no linezolid intervention’ control group) conducted by Tang et al. [51] in China was identified with a total sample size of 65 patients. This is the first of such kind compared to the randomized trials reported by Lee et al. [42] where both study groups were administered linezolid.

### Efficacy

In our review, linezolid was administered in combination with other anti-tubercular drugs to achieve treatment success. Thus treatment success may not be exclusively attributed to linezolid. Nonetheless, since linezolid inclusion mostly followed resistance or treatment failure with other second line drugs, much of the treatment success may be attributed to linezolid. We obtained a pooled culture conversion of 88.45 % (95 % CI 83.82–92.38 %, p = 0.0112). Previous reviews by Sotgui et al. [22] and Zhang et al. [23] obtained pooled culture conversion of 93 % (p = 0.2704) and 89 % (p = 0.0217) respectively. The results from our study shows strong evidence (p = 0.0112) of linezolid to achieve culture conversion in MDR/XDRTB patients which is synonymous to that from Zhang et al. (p = 0.0217) [23] due to large samples size in these studies compared to Sotgui et al. [22] whose results depicted otherwise.

On the other hand, pooled treatment success was significantly lower [77.36 % (95 % CI 71.38–82.83 %, p = 0.0365)] than that obtained for culture conversion. This is also similar to the stated cure rates in the 2015 WHO Global TB report and also results obtained from previous reviews [1, 19, 22, 23]. The 2015 WHO Global report on TB, reports cure rates in 2014 from 43 countries as ≥ 75 % with global average cure rate of 50 %. The treatment success proportion obtained in our review in comparison with the culture conversion significantly implies that, most of the MDR/XDR TB patients who achieve sputum culture conversion do not achieve treatment success. This may be due to default, treatment failure, treatment discontinuation due to adverse effects or relapse. In a study conducted by Xu et al. [56], all patients (n = 18) administered linezolid were culture negative at 7 weeks of treatment during hospital admission. However, at data censor after patient discharge from the hospital, only nine patients (50 %) achieved treatment success whiles three and two patients relapsed and attained treatment failure, respectively. Synonymously, five studies [42, 43, 49, 55, 56] which reported patient admission prior to linezolid administration and discharge after culture conversion also observed higher proportions of culture conversion than treatment success.

Fifteen studies (Table 2) did not report on hospitalization whiles two studies initiated linezolid under outpatient environment [47, 53]. One out of these two studies obtained a lower treatment success (61.10 %) compared to the other [47, 53]. These results contribute to the significance of hospitalization prior to treatment initiation in MDR/XDR TB which enhances therapeutic and adverse events monitoring as well as patient compliance to therapy to achieve high proportions of treatment success. Nonetheless, Roongruangpitayakul et al. [47] reports high treatment success proportion (88.2 %) obtained under outpatient conditions. This may imply that with efficient DOT, patients not requiring hospitalization can also be successfully treated with linezolid as long as procedures are in place to monitor incidence of adverse events.

Administering different doses of linezolid did not show any significant difference in culture conversion and treatment success; p = 0.0948 and p = 0.0695, respectively. A recent systematic review conducted by Zhang et al. also had similar results for culture conversion and favorable outcomes, respectively [23]. Thus, linezolid may be administered at a lower dose to achieve treatment success whiles reducing the incidence of adverse events. However, the dose and duration of linezolid in the treatment of MDR/XDR TB ought to be streamlined based on evidence from randomized controlled trials (RCTs). An RCT conducted by Tang et al. and involving 65 XDR-TB patients reported higher proportions of culture conversion (96 vs. 41 %) and treatment success (79.31 vs. 37.93 %) in the treatment group than the control group [51]. Also in this same RCT, patients were given an initial high dose of linezolid (1200 mg daily) for a period of 4–6 weeks followed by a reduced dose (300–600 mg) in the continuous phase to complete a 24 month treatment regimen. This may propose a successful dosage regimen for MDR/XDR TB involving a maximum tolerable high dose of 1200 mg daily for a short intensive phase, followed by a reduced dose between 300 and 600 mg daily during the continuous phase. Nonetheless, RCTs involving larger patient population need to be conducted to strengthen this evidence. Horsburg et al. proposes a novel method to ascertain optimum duration of antibiotic treatment regimen [58]. Their proposed model utilizes a logistic regression model in RCT design to ascertain the shortest possible antibiotic treatment duration in relation to corresponding proportions of cured patients. The researchers further highlighted the suitability of this proposed model for anti-tubercular regimen with the aim of minimizing the incidence of resistance, toxicity, costs and pill burden [58].

### Safety and tolerability

The major adverse events identified in this review were neuropathy and myelosuppression. Other minor adverse effects which were predominantly gastrointestinal related including nausea and vomiting were reported with a minimum pooled proportion of 33.60 % (95 % CI = 20.41–48.23 %). Only one case of rhabdomyolysis has been reported by Lee et al. [42] which is an important observation to note in case of an emerging rare adverse effects of linezolid. Myelosuppression occurred at a higher proportion than neuropathy with both adverse events bearing a significant association with linezolid with combined proportions of 32.93 and 29.92 % respectively (p < 0.0001). In most studies, myelosuppression and neuropathy effects were managed by temporarily or permanently (15.01 %, p < 0.0001) discontinuing linezolid therapy. However, in some patients, incidence of severe anemia was remedied by blood transfusion [37]. Two cases were reported by Von der Lippe et al. for attaining normal full blood count upon withdrawal of linezolid without having to undergo blood transfusion [55]. The incidence of myelosuppression was significantly dose related (p < 0.0001) with lower doses associated with lower incidence. The incidence of neuropathy was reported in all studies except one [39]. Roongruangpitayakul et al. observed reversible optic neuropathy and irreversible peripheral neuropathy in patients who suffered these effects following treatment discontinuation and administration of vitamin B supplement [47]. Persistent irreversible neuropathy has also been previously reported by two other studies [53, 55]. From the results obtained (p = 0.52), neuropathy was not strongly associated with higher doses of linezolid and as such close monitoring of patients is encouraged irrespective of the dose administered. Therefore, in order to improve tolerability of linezolid regimen in MDR/XDR TB, a combined high dose (1200 mg daily) aimed at a shorter duration and lower dose (300–600 mg) targeted at a longer continuous phase may be employed with effective patient monitoring to inform dose adjustments when required.

### Strengths and limitations

The major strength of our study is the large patient population (n = 507) which depicts a more significant and stronger evidence compared to previous reviews which included patient population of 218 [19], 121 [22] and 239 [23]. However, there are limitations including the higher proportion of non-randomized case series and retrospective studies (n = 21). This increases the likelihood of reporting and selection bias. Additionally, significant heterogeneity among studies are evident including presence of publication bias. Moreover, while this review provides some useful understanding regarding the safety and efficacy of linezolid, only 3 % of the patients involved in the studies’ reviewed had documented HIV positive status. This calls for further research targeted at assessing the efficacy and safety of linezolid in HIV patients as they are more likely to develop active TB and TB-related mortality rates among them remains higher than the general population [59]. Also, better data would be needed to evaluate for instance treatment duration that optimally balances favorable clinical outcomes but minimizes occurrence of adverse effects to improve patient safety. Furthermore, while an earlier RCT conducted by Padayatchi et al. [60] was challenged by patient recruitment and retention, this has been overcome by a successfully conducted RCT by Tang et al. [51] while the sample size was relatively small, results from this RCT showed significant treatment success in the treatment group compared to the control group (*p* = 0.013). Nevertheless, there is urgent need for highly powered RCTs with larger sample size across highly endemic regions including participants from Africa to better inform the magnitude and significance of linezolid treatment effect in MDR and XDR TB patients.

## Conclusions

Evidence available mainly from observational studies has demonstrated linezolid to be effective in the treatment of MDR/XDR TB. This presents the drug as a viable option towards effective pharmacotherapy for MDR/XDR TB which is increasingly becoming a global health challenge. Nonetheless, patients ought to be monitored closely for the incidence of major adverse events such as myelosuppression and neuropathy. To minimize adverse effects and improve clinical outcomes, a combined high dose (1200 mg daily) for an intensive phase followed by a lower dose (300–600 mg daily) for a continuous phase is proposed along with effective patient monitoring to inform dose adjustments when required. This may however require thorough future research investigation. Specific TB guidelines incorporating the use of linezolid are required and wider commitments from all global health players are needed to address barriers such as the high cost of the drug if successful use and accessibility is to be achieved particularly in low-resourced settings where majority of TB patients live.

## Additional file

10.1186/s12941-016-0156-y Studies extracted data for outcomes evaluation.

## Abbreviation

AIDS: acquired immune deficiency syndrome
HIV: human immunodeficiency virus
PRISMA: preferred reporting items for systematic reviews and meta-analyses
WHO: World Health Organization
DOT: directly observed treatment
TB: tuberculosis
MDR: multi-drug resistant
XDR: extensively-drug resistant
DST: drug sensitivity testing
RCT: randomized controlled trial
IRB: Institutional Review Board

## References

[1] WHO. Global tuberculosis report 2015. http://www.apps.who.int/iris/bitstream/10665/191102/1/9789241565059_eng.pdf?ua=1 Accessed 2 Feb 2016.

[2] Maartens G, Wilkinson RJ (2007). Tuberculosis. Lancet.

[3] WHO. Tuberculosis. http://www.who.int/mediacentre/factsheets/fs104/en/ Accessed 2 Feb 2016.

[4] WHO. Tuberculosis: the global burden 2005. http://www.who.int/tb/publications/tb_global_facts_sep05_en.pdf Accessed 2 Feb 2016.

[5] Reid A, Grant AD, White RG, Dye C, Vynnycky E, Fielding K, Churchyard G, Pillay Y (2015). Accelerating progress towards tuberculosis elimination: the need for combination treatment and prevention. Int J Tuberc Lung Dis.

[6] WHO (2006). Extensively drug-resistant tuberculosis (XDR-TB): recommendations for prevention and control. Wkly Epidemiol Rec.

[7] WHO. Multidrug-resistant tuberculosis (MDR-TB). http://www.who.int/tb/challenges/mdr/en/ Accessed 2 Feb 2016.

[8] Matteelli A, Roggi A, Carvalho AC (2014). Extensively drug-resistant tuberculosis: epidemiology and management. Clin Epidemiol.

[9] WHO. Multi-drug resistant tuberculosis (MDR-TB): 2015 update http://www.who.int/tb/challenges/mdr/mdr_tb_factsheet.pdf Accessed 3 Feb 2016.

[10] Diel R, Nienhaus A, Lampenius N, Rüsch-Gerdes S, Richter E (2014). Cost of multi drug resistance tuberculosis in Germany. Respir Med.

[11] StopTBPartnership. Global plan to stop TB, 2011–2015 http://www.stoptb.org/global/plan/plan1115.asp Accessed 9 Feb 2016.

[12] WHO (2011). Towards universal access to diagnosis and treatment of multidrug-resistant and extensively drug resistant tuberculosis by 2015: WHO progress report 2011.

[13] Alffenaar JWC, van Altena R, Harmelink IM, Filguera P, Molenaar E, Wessels AMA (2010). Comparison of the pharmacokinetics of two dosage regimens of linezolid in multidrug-resistant and extensively drug-resistant tuberculosis patients. Clin Pharmacokinet.

[14] Sotgiu G, Pontali E, Migliori GB (2015). Linezolid to treat MDR-/XDR-tuberculosis: available evidence and future scenarios. Eur Respir J.

[15] Field SK, Fisher D, Jarand JM (2012). New treatment options for multidrug-resistant tuberculosis. Ther Adv Respir Dis.

[16] Alcala L, Ruiz-Serrano JM, Turegano CP, de Viedma GD, Diaz-Infantes M, Marin-Arriaza M (2003). In vitro activities of linezolid against clinical isolates of *Mycobacterium tuberculosis* that are susceptible or resistant to first-line antituberculous drugs. Antimicrob Agents Chemother.

[17] Guna R, Munoz C, Dominguez V, Garcia-Garcia A, Galvez J, de Julian-Ortiz J (2005). In vitro activity of linezolid, clarithromycin and moxifloxacin against clinical isolates of Mycobacterium kansasii. J Antimicrob Chemother.

[18] Yang C, Hong L, Wang D, Meng X, He J, Tong A (2012). In vitro activity of linezolid against clinical isolates against *Mycobacterium tuberculosis*, including multi-drug resistant and extensively drug-resistant strains from Beijing, China. Jpn J Infect Dis.

[19] Cox H, Ford N (2012). Linezolid for the treatment of complicated drug-resistant tuberculosis: a systematic review and meta-analysis. Int J Tuberc Lung Dis.

[20] Jaramillo E, Weyer K, Raviglione M (2013). Linezolid for extensively drug-resistant tuberculosis. N Engl J Med.

[21] Agyeman A, Ofori-Asenso R. Linezolid for the treatment of multi-drug and extensively drug resistant tuberculosis: a systematic review on efficacy and toxicity. Internet J Pharmacol. 2014;13(1).

[22] Sotgiu G, Centis R, D’Ambrosio L, Alffenaar JW, Anger HA, Caminero JA, Castiglia P, De Lorenzo S, Ferrara G, Koh WJ, Schecter GF, Shim TS, Singla R, Skrahina A, Spanevello A, Udwadia ZF, Villar M, Zampogna E, Zellweger JP, Zumla A, Migliori GB (2012). Efficacy, safety and tolerability of linezolid containing regimens in treating MDR-TB and XDR-TB: systematic review and meta-analysis. Eur Respir J.

[23] Zhang X, Falagas ME, Vardakas KZ, Wang R, Qin R, Wang J, Liu Y (2015). Systematic review and meta-analysis of the efficacy and safety of therapy with linezolid containing regimens in the treatment of multidrug-resistant and extensively drug-resistant tuberculosis. J Thorac Dis.

[24] Whitlock EP, Lin JS, Chou R, Shekelle P, Robinson KA (2008). Using existing systematic reviews in complex systematic reviews. Ann Intern Med.

[25] Moher D, Liberati A, Tetzlaff J, Altman DG (2009). Preferred reporting items for systematic reviews and meta-analyses: the PRISMA statement. Ann Intern Med.

[26] Wikipedia. Linezolid. https://www.en.wikipedia.org/wiki/Linezolid Accessed 2 Feb 2016.

[27] Chan K, Bhandari M (2011). Three-minute critical appraisal of a case series article. Indian J Orthop.

[28] Law M, Stewart D, Pollock N, Letts L, Bosh J, Westmorland M. Critical review form-quantitative studies. McMaster University 1998 (28th July, 1998). http://www.srs-mcmaster.ca/wp-content/uploads/2015/04/Critical-Review-Form-Quantitative-Studies-English.doc. Accessed 1 Jan 2016.

[29] Deenadayalan Y, Perraton L, Machotka Z, Kumar S (2010). Day therapy programs for adolescents with mental health problems: a systematic review. Internet J Allied Health Sci Pract.

[30] WHO. Treatment of tuberculosis: guidelines 2010 http://www.whqlibdoc.who.int/publications/2010/9789241547833_eng.pdf. Accessed 2 Feb 2016.

[31] TBOnline. Linezolid. http://www.tbonline.info/posts/2011/8/24/linezolid/ Accessed 2 Feb 2016.

[32] StatsDirect. Proportion meta-analysis http://www.statsdirect.com/help/default.htm#meta_analysis/proportion.htm. Accessed 4 February 2016.

[33] Higgins JPT, Thompson SG, Deeks JJ, Altman DG (2003). Measuring inconsistency in Meta-analyses. Br Med J.

[34] Song F, Khan KS, Dinnes J, Sutton AJ (2002). Asymmetric funnel plots and publication bias in meta-analyses of diagnostic accuracy. Int J Epidemiol.

[35] Abbate E, Vescovo M, Natiello M, Cufre M, Garcia A, Gonzalez Montaner P (2012). Successful alternative treatment of extensively drug-resistant tuberculosis in Argentina with a combination of linezolid, moxifloxacin and thioridazine. J Antimicrob Chemother.

[36] Anger HA, Dworkin F, Sharma S, Munsiff SS, Nilsen DM, Ahuja SD (2010). Linezolid use for treatment of multidrug-resistant and extensively drug-resistant tuberculosis, New York City, 2000–2006. J Antimicrob Chemother.

[37] Condos R, Hadgiangelis N, Leibert E, Jacquette G, Harkin T, Rom WN (2008). Case series report of a linezolid-containing regimen for extensively drug-resistant tuberculosis. Chest.

[38] De Lorenzo S, Centis R, D’Ambrosio L, Sotgiu G, Migliori GB (2012). On linezolid efficacy and tolerability. Eur Respir J.

[39] Fortún J, Martín-Dávila P, Navas E, Pérez-Elías MJ, Cobo J, Tato M, De la Pedrosa EG, Gómez-Mampaso E, Moreno S (2005). Linezolid for the treatment of multidrug-resistant tuberculosis. J Antimicrob Chemother.

[40] Koh WJ, Kwon OJ, Gwak H, Chung JW, Cho SN, Kim WS (2009). Daily 300 mg dose of linezolid for the treatment of intractable multidrug-resistant and extensively drug-resistant tuberculosis. J Antimicrob Chemother.

[41] Koh WJ, Kang YR, Jeon K, Jung Kwon O, Lyu J, Kim WS (2012). Daily 300 mg dose of linezolid for multidrug-resistant and extensively drug-resistant tuberculosis: updated analysis of 51 patients. J Antimicrob Chemother.

[42] Lee M, Lee J, Carroll MW, Choi H, Min S, Song T (2012). Linezolid for treatment of chronic extensively drug-resistant tuberculosis. N Engl J Med.

[43] Liu Y, Bao P, Wang D, Li Y, Tang L, Zhou Y, Zhao W (2015). Clinical outcomes of linezolid treatment for extensively drug-resistant tuberculosis in Beijing, China: a hospital-based retrospective study. Jpn J Infect Dis.

[44] Migliori GB, Eker B, Richardson MD, Sotgiu G, Zellweger JP, Skrahina A, Ortmann J, Girardi E, Hoffmann H, Besozzi G, Bevilacqua N, Kirsten D, Centis R, Lange C, TBNET Study Group (2009). A retrospective TBNET assessment of linezolid safety, tolerability and efficacy in multidrug-resistant tuberculosis. Eur Respir J.

[45] Nam HS, Koh WJ, Kwon OJ, Cho SN, Shim TS (2009). Daily half-dose linezolid for the treatment of intractable multidrug-resistant tuberculosis. Int J Antimicrob Agents.

[46] Park IN, Hong SB, Oh YM, Kim MN, Lim CM, Lee SD (2006). Efficacy and tolerability of daily-half dose linezolid in patients with intractable multidrug-resistant tuberculosis. J Antimicrob Chemother.

[47] Roongruangpitayakul C, Chuchottaworn C (2013). Outcomes of MDR/XDR-TB patients treated with linezolid: experience in Thailand. J Med Assoc Thai.

[48] Schecter GF, Scott C, True L, Raftery A, Flood J, Mase S (2010). Linezolid in the treatment of multidrug-resistant tuberculosis. Clin Infect Dis.

[49] Singla R, Caminero JA, Jaiswal A, Singla N, Gupta S, Bali RK (2012). Linezolid: an effective, safe and cheap drug for patients failing multidrug-resistant tuberculosis treatment in India. Eur Respir J.

[50] Tang SJ, Zhang Q, Zeng LH, Sun H, Gu J, Hao XH (2011). Efficacy and safety of linezolid in the treatment of extensively drug-resistant tuberculosis. Jpn J Infect Dis.

[51] Tang S, Yao L, Hao X, Zhang X, Liu G, Liu X, Wu M, Zen L, Sun H, Liu Y, Gu J, Lin F, Wang X, Zhang Z (2015). Efficacy, safety and tolerability of linezolid for the treatment of XDR-TB: a study in China. Eur Respir J.

[52] Tse-Chang A, Kunimoto D, Der E, Ahmed R (2013). Assessment of linezolid efficacy, safety and tolerability in the treatment of tuberculosis: a retrospective case review. Can J Infect Dis Med Microbiol.

[53] Udwadia ZF, Sen T, Moharil G (2010). Assessment of linezolid efficacy and safety in MDR- and XDR-TB: an Indian perspective. Eur Respir J.

[54] Villar M, Sotgiu G, D’Ambrosio L, Raymundo E, Fernandes L, Barbedo J (2011). Linezolid safety, tolerability and efficacy to treat multidrug- and extensively drug-resistant tuberculosis. Eur Respir J.

[55] Von Der Lippe B, Sandven P, Brubakk O (2006). Efficacy and safety of linezolid in multidrug resistant tuberculosis (MDR-TB)—a report of ten cases. J Infect.

[56] Xu HB, Jiang RH, Li L, Xiao HP (2012). Linezolid in the treatment of MDR-TB: a retrospective clinical study. Int J Tuberc Lung Dis.

[57] Zhang L, Pang Y, Yu X, Wang Y, Gao M, Huang H, Zhao Y (2014). Linezolid in the treatment of extensively drug-resistant tuberculosis. Infection.

[58] Horsburgh CR, Shea KM, Phillips P, Lavalley M (2013). Randomized clinical trials to identify optimal antibiotic treatment duration. Trials.

[59] CDC. Drug-resistant TB. http://www.cdc.gov/tb/topic/drtb/ Accessed May 5 2016.

[60] Padayatchi N, Mac Kenzie WR, Hirsch-Moverman Y, Feng PJ, Villarino E, Saukkonen J, Heilig CM, Weiner M, El-Sadr WM (2012). Lessons from a randomized clinical trial for multidrug-resistant tuberculosis. Int J Tuberc Lung Dis.

