# Supplementary material for: Efficacy and safety profile of linezolid in the treatment of multidrug-resistant (MDR) and extensively drug-resistant (XDR) tuberculosis: a systematic review and meta-analysis
Source: Ann Clin Microbiol Antimicrob. 2016 Jun 22;15:41. doi: 10.1186/s12941-016-0156-y (PMC4917997; doi:10.1186/s12941-016-0156-y)
Supplement: Supplementary file 1 — 10.1186/s12941-016-0156-y Studies extracted data for outcomes evaluation. [file 12941_2016_156_MOESM1_ESM.docx]

| **Reference** | **Outcomes reported** | | | | | | | |
| --- | --- | --- | --- | --- | --- | --- | --- | --- |
|  | **Evaluable outcomes for Culture Conversion** | **No. Sputum Culture converters** | **Evaluable outcomes for Treatment Success** | **No. achieved Treatment Success** | **No.**  **(Myelosuppression)** | **No.**  **(Neuropathy)** | **Discontinuation due to LZD adverse effects** | **No.**  **(Any other adverse events)** |
| Abbate et al. (2012) | 15 | 15 | 15 | 15 | 4 | 3 | 0 | 2 |
| Anger et al. (2010) | 11 | 11 | 16 | 11 | 13 | 7 | 5 | 16 |
| Condos et al. (2008) | 6 | 5 | 6 | 6 | 3 | 2 | 0 | 0 |
| De Lorenzo et al. (2012) | 12 | 9 | 3 | 1 | 2 | 2 | 0 | 0 |
| Fortun et al. (2005) | 3 | 3 | 2 | 1 | 2 | 0 | 1 | 2 |
| Koh et al. (2009) | 24 | 22 | 6 | 4 | 1 | 8 | 1 | 9 |
| Koh et al. (2012) | 45 | 34 | 45 | 34 | 10 | 14 | 14 | 0 |
| Lee et al. (2012) | 38 | 34 | 17 | 13 | 7 | 28 | 3 | 1 |
| Liu et al (2015) | 16 | 14 | 13 | 11 | 6 | 3 | 2 | 5 |
| Migliori et al. (2009) | 45 | 39 | 45 | 36 | 23 | 3 | 19 | 35 |
| Nam et al. (2009) | 11 | 9 | 11 | 6 | 2 | 9 | 9 | 9 |
| Park et al.(2006) | 8 | 8 | 3 | 1 | 1 | 4 | 2 | 6 |
| Roongruangpitayakul et al (2013) | 24 | 22 | 17 | 15 | 3 | 5 | 5 | 2 |
| Schecter et al. (2010) | 29 | 29 | 25 | 22 | 2 | 6 | 3 | 9 |
| Singla et al. (2012) | 29 | 23 | 14 | 9 | 10 | 5 | 3 | 16 |
| Tang et al. (2011) | 14 | 14 | 14 | 11 | 6 | 3 | 0 | 4 |
| Tang et al. (2015) | 29 | 26 | 29 | 23 | 26 | 14 | 2 | 16 |
| Tse-Chang et al. (2013) | 13 | 11 | 9 | 9 | 11 | 1 | 3 | 2 |
| Udwadia et al. (20) | 18 | NR | 18 | 11 | 1 | 7 | NR | 11 |
| Villar et al.(2011) | 9 | 9 | 9 | 8 | 2 | 1 | NR | 0 |
| Von der Lippe et al. (2006) | 9 | 9 | 10 | 9 | 5 | 6 | 7 | NR |
| Xu et al. (2012) | 14 | 12 | 11 | 9 | 13 | 11 | 1 | 15 |
| Zhang et al. (2014) | 15 | 9 | 15 | 9 | 4 | 1 | 0 | 5 |

NR= Not Reported

**Table X**: Studies Extracted Data for Outcomes evaluation.
